# Increasing Fluroquinolone Susceptibility and Genetic Diversity of ESBL-Producing *E. coli* from the Lower Respiratory Tract during the COVID-19 Pandemic

**DOI:** 10.3390/antibiotics13090797

**Published:** 2024-08-23

**Authors:** Katja Hrovat, Katja Seme, Jerneja Ambrožič Avguštin

**Affiliations:** 1 Department of Biology, Biotechnical Faculty, University of Ljubljana, 1000 Ljubljana, Slovenia; katja.hrovat@bf.uni-lj.si; 2 Institute of Microbiology and Immunology, Faculty of Medicine, University of Ljubljana, 1000 Ljubljana, Slovenia; katja.seme@mf.uni-lj.si

**Keywords:** antimicrobial resistance, *Escherichia coli*, extended-spectrum β-lactamases, lower respiratory tract, COVID-19

## Abstract

Lower respiratory tract infections (LRTIs) are the fourth leading cause of death worldwide, among which *Escherichia coli* (*E. coli*) pneumonia is considered a rare phenomenon. Treatment options for LRTIs have become limited, especially for extended-spectrum β-lactamase-producing *E. coli* (ESBL-EC), which are usually resistant to other groups of antimicrobials as well. The aim of our study was to compare the phenotypic resistance profiles and genotypes of ESBL-EC isolates associated with LRTIs before (pre-COVID-19) and during (COVID-19) the COVID-19 pandemic. All isolates were screened for antimicrobial resistance genes (ARGs) and virulence-associated genes (VAGs) and assigned to phylogenetic groups, sequence types and clonal groups by PCR. During the pandemic, a significantly lower proportion of ciprofloxacin-, levofloxacin- and trimethoprim-sulfamethoxazole-resistant ESBL-EC isolates was retrieved from lower respiratory tract (LRT) samples. PCR-based genotypization revealed greater clonal diversity and a significantly lower proportion of isolates with *bla*_TEM_, *aac(6′)-Ib-cr* and *qacEΔ1* genes. In addition, a higher proportion of isolates with the integrase gene *int1* and virulence genes *sat* and *tsh* was confirmed. The lower prevalence of fluoroquinolone resistance and greater genetic diversity of ESBL-EC isolated during the COVID-19 period may have been due to the introduction of new bacterial strains into the hospital environment, along with changes in clinical establishment guidelines and practices.

## 1. Introduction

In 2017, the World Health Organization (WHO) placed *Enterobacteriaceae*, including ESBL-producing *E. coli*, on the priority list in the search for new antimicrobial compounds [1]. In addition, a systematic analysis by the Antimicrobial Resistance Collaborators estimated that 7.7 million deaths (13.6%) in 2019, the year before the SARS-CoV-2 outbreak, were due to bacterial infections, and that five bacterial pathogens, including *E. coli*, were responsible for more than half of all global bacterial deaths in 2019, contributing to the burden of antimicrobial resistance (AMR) [2]. *Escherichia coli* (*E. coli*), a ubiquitous Gram-negative bacterium, can acquire and transmit antimicrobial resistance genes (ARGs) and is often associated with intestinal and extraintestinal diseases [3]. Third-generation cephalosporins, fluoroquinolones (FQs) and a combination of trimethoprim and sulfamethoxazole (SXT) are frequently used for the effective treatment of bacterial infections caused by *E. coli* [1]. However, the treatment of these infections is becoming progressively difficult due the increasing prevalence of ESBL-producing strains, which are often associated with resistance to several antimicrobial classes, including FQs, SXT and even last-resort antimicrobials such as carbapenems [1,4]. ESBL-EC is capable of hydrolyzing third-generation cephalosporins and monobactams, but its activity is inhibited by clavulanic acid and tazobactam. It is often associated with multidrug resistance to other antimicrobial classes [4]. Genes encoding ESBLs and SXT resistance frequently reside on conjugative plasmids, enabling their spread to different strains and consequently leading to a variation in the gene composition of the bacterial population. On the contrary, clinically significant FQ resistance primarily arises from mutations of the chromosomal genes encoding DNA gyrase and topoisomerase IV, the target enzymes of fluoroquinolones, or the efflux pumps [5,6]. Due to the tendency to develop resistance and their adverse effects, the use of FQs as a first-line treatment of severe community-acquired pneumonia and complicated urinary tract infections (UTIs) is therefore restricted, despite their broad-spectrum activity [7,8,9,10].

The spread of ESBL-EC accelerated after 2003, when successful pandemic clones such as *E. coli* ST131, equipped with a combination of virulence and resistance genes, including for plasmid-mediated quinolone resistance (PMQR), also significantly contributed to the increase in fluoroquinolone resistance rates, rendering FQ treatment options ineffective [11]. For the past 30 years, SXT has played a crucial role in treating various infections. Although resistance has increased, and newer antimicrobials have been introduced, SXT remains valuable when considering resistance patterns and clinical factors. It is still a cost-effective choice for targeted therapy, potentially slowing the development of resistance to newer antimicrobials [12]. Due to its worldwide distribution, ESBL-EC has been the subject of extensive research. However, most studies have been conducted on isolates from urinary tract or blood cultures, while little is known about isolates from the respiratory tract.

During the COVID-19 pandemic, medical practices were changed, leading to in-creased consumption of antimicrobials, including disinfectants, in many healthcare institutions [13,14,15]. Therefore, an increased rate of antimicrobial resistance could be expected.

Our previous study analyzing ESBL-EC from LRTs over an 18-year period showed the persistence of certain strains with the same ERIC-PCR profile in the Central Slovenia region, indicating the presence of hospital-adapted (clonal) strains, with the ST131 sequence group being the most prevalent one [16]. Although several studies have aimed to determine the impact of the COVID-19 period on the resistance rates of bacteria isolated from various samples in different regions recently, the dynamics of AMR during this period remain uncertain [17,18]. Therefore, our present study aimed to analyze the phenotypic resistance and selected genetic marker genes, including ARGs and VAGs, of ESBL-EC isolated from LRTs in the pandemic years of 2020–2022 and compare the results with data for isolates from the pre-pandemic period (2017–2019) [14,15]. This comparison may help to determine whether the COVID-19 pandemic and associated changes in the healthcare system have affected the AMR and population structure of ESBL-EC.

## 2. Results

### 2.1. LRT ESBL-EC Isolates from Both Study Periods

In this study, 119 ESBL-EC samples isolated in the pre-COVID-19 period were compared to 81 ESBL *E. coli* samples isolated during the COVID-19 period. The clinical samples were obtained from 70 (58.8%) male and 49 (41.2%) female patients in the pre-COVID-19 period and from 57 (70.4%) male and 24 (29.6%) female patients in the COVID-19 period (Table 1). In the pre-COVID-19 period, 87 (73.1%), 90 (25.2%) and 2 (1.7%) strains were isolated from tracheal aspirates, sputa and bronchoalveolar lavages, respectively, while in the COVID-19 period, 50 (61.7%), 21 (25.9%) and 10 (12.3%) strains were isolated from tracheal aspirates, sputa and bronchoalveolar lavages, respectively. The average age of the patients in the pre-COVID-19 period was 67.3 years, and it was 70.8 years for the patients in the COVID-19 period. In the pre-COVID-19 period, the majority of patients belonged to the age groups 81–90 years (n = 36; 30.3%), 71–80 years (n = 28; 23.5%), 61–70 years (n = 20; 16.8%), 0–10 years (n = 11; 9.2%) and 91 years and older (n = 8; 6.7%), while the majority of patients in the COVID-19 period belonged to the age groups 81–90 years (n = 27; 33.3%), 71–80 years (n = 20; 24.7%), 61–70 years (n = 18; 22.2%), 41–50 years (n = 5; 6.2%) and 51–60 years (n = 5; 6.2%). In all, 99 (83.2%) and 72 (88.9%) patients were older than 51 years in the pre-COVID-19 and COVID-19 periods, respectively.

### 2.2. Antimicrobial Resistance of ESBL-EC from Both Study Periods

**Figure 1:**
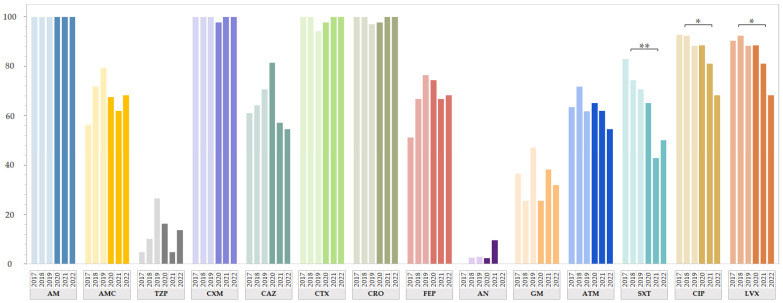
Resistance rate of ESBL-EC from LRT isolated between 2017 and 2022. Lighter shades represent the prevalence of resistant isolates before the COVID-19 pandemic, and darker shades are for resistant isolates during the COVID-19 pandemic. Statistical differences between the two time periods (pre-COVID-19 and COVID-19) were marked as significant (* *p* < 0.05) or as highly significant (** *p* < 0.001). Data for carbapenems (ertapenem, imipenem and meropenem) are not shown as only one resistant isolate was detected. Abbreviations for antimicrobial agents: AM = ampicillin; AMC = amoxicillin-clavulanic acid; TZP = piperacillin-tazobactam; CXM = cefuroxime; CAZ = ceftazidime; CTX = cefotaxime; CRO = ceftriaxone; FEP = cefepime; AN = amikacin; GM = gentamicin; ATM = aztreonam; SXT = trimethoprim-sulphametoxazole; CIP = ciprofloxacin; LVX = levofloxacin.

The resistance rates for 14 tested antimicrobials from both study periods are shown in Figure 1 by year of isolation. To summarize, of the 119 ESBL-EC isolates isolated from the LRT in the pre-COVID-19 period, 119 (100%) showed resistance to AM, 91 (76.5%) showed resistance to SXT, 82 (68.9%) showed resistance to AMC, 78 (65.5%) showed resistance to ATM, and 16 (13.4%) showed resistance to TZP (Table S4 in Supplementary Materials). High percentages of resistance to cephalosporins (64.7–100%) and fluoroquinolones (≈91%) were detected. In contrast, low resistance rates were found for aminoglycosides (1.7% to AN and 35.3% to GM), and none of the isolates were resistant to carbapenems. The antimicrobial resistance rates of 81 ESBL-EC isolates recovered from the LRT in the COVID-19 period were similar, as 81 (100%) showed resistance to AM, 53 (65.4%) showed resistance to AMC, 50 (61.7%) showed resistance to ATM, and 10 (12.3%) showed resistance to TZP. While the resistance rates for cephalosporins in the COVID-19 period ranged between 67.9% and 98.8%, the resistance rates to fluoroquinolones (80.2%) and SXT (54.3%) were significantly lower compared with the pre-COVID-19 period. In addition, low resistance rates to aminoglycosides (3.7% to AN and 30.9% to GM) and carbapenems were observed, as only one isolate (1.2%) was resistant to ETP. All isolates from both study periods were defined as MDR, as they were not susceptible to at least one agent from three or more antimicrobial categories.

**Figure 2:**
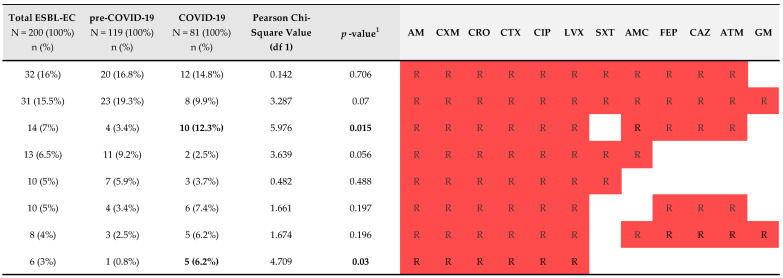
Prevalence of resistance profiles of ESBL-EC isolates from LRT. Red colored R indicated resistance to antimicrobial agents, and the bold numbers indicate statistically significant differences between the two study periods. The antimicrobials were ordered according to the prevalence of their susceptibility (from least to most susceptible). The antimicrobial agents TZP, AN, ETP, IPM and MEM were not included in the scheme as the prevalence of resistance to these antimicrobial agents was extremely low (<13%). ^1^ Statistical differences between the two time periods (pre-COVID-19 and COVID-19) were considered significant (*p* < 0.05) or highly significant (*p* < 0.001), and these are marked in bold. Abbreviations for antimicrobial agents: AM = ampicillin; AMC = amoxicillin-clavulanic acid; CXM = cefuroxime; CAZ = ceftazidime; CTX = cefotaxime; CRO = ceftriaxone; FEP = cefepime; GM = gentamicin; ATM = aztreonam; SXT = trimethoprim-sulphametoxazole; CIP = ciprofloxacin; LVX = levofloxacin.

The most prevalent resistance profile in the pre-COVID-19 period was AM-CXM-CRO-CTX-CIP-LVX-SXT-AMC-FEP-CAZ-ATM-GM (n = 23; 19.3%), which also applied to isolates from the COVID-19 period, except that the latter were susceptible to GM (n = 12; 14.8%) (Figure 2 and Table S5 in Supplementary Materials).

The majority of the isolates from the pre-COVID-19 period were resistant to 12 (n = 22; 18.5%), 11 (n = 22; 18.5%) and 8 (n = 22; 18.5%) out of 17 of the antimicrobials tested, while the majority (n = 15; 18.5%) of the isolates from the COVID-19 period were resistant to 11 out of the 17 antimicrobials tested, followed by 9 out of 17 (n = 12; 14.8%), 10 out of 17 (n = 10; 12.3%) and 8 out of 17 (n = 10; 12.3%) of the antimicrobials tested, respectively. One isolate from the COVID-19 period showed resistance to 14 out of 17 of the antimicrobial agents tested.

### 2.3. Comparison of Genotype Data of ESBL-EC Isolated from LRTs from Both Study Periods

A comparison of the β-lactamase group genes showed that *bla*_CTX-M-1_ was detected most frequently in both study periods (84.9% pre-COVID-19 and 75.3% during COVID-19), while the frequency of the *bla*_TEM_ gene was statistically significantly higher in the pre-COVID-19 period (52.9% pre-COVID-19 versus 32.1% during COVID-19; *p* = 0.004) (Table 2). This also applied to the plasmid-mediated fluoroquinolone-acetylating aminoglycoside-(6)-N-acetyltransferase (*aac[6′]-I-cr*) gene (38.7% pre-COVID-19 versus 24.7% during COVID-19; *p* = 0.039) and the biocide resistance gene *qacEΔ1* (52.1% pre-COVID-19 versus 29.6% during COVID-19; *p* = 0.002) (Table 2). In contrast, a significantly higher proportion of isolates with the integron-associated *int1* gene (27.7% pre-COVID-19 versus 45.7% during COVID-19; *p* = 0.009) was detected during the COVID-19 pandemic (Table 2).

Clonal diversity, according to the ERIC-PCR profiles, phylogenetic group and sequence type assignment and presence of selected VAGs, revealed different ESBL-EC population structures in the pre-COVID-19 and COVID-19 periods. ESBL-EC from LRTs isolated during the COVID-19 pandemic were more evenly assigned to ERIC profile groups compared with the isolates from the pre-COVID-19 period (Table 3). While the most prevalent ERIC profile groups in the pre-COVID-19 period were EP1 (n = 54; 45.4%), EP2 (n = 8; 6.7%) and EP3 (n = 21; 17.6%), with the first two groups also being statistically significantly related to the pre-COVID-19 period (*p* = 0.001 for EP1 and *p* = 0.017 for EP2), in the COVID-19 period, the most prevalent groups were EP3 (n = 23; 28.4%), EP1 (n = 18; 22.2%) and EP4 (n = 9; 11.1%). Isolates with unique profiles or clusters of isolates with a number of less than five were assigned to EPx groups. Although there were no statistical differences, more isolates from the COVID-19 period were included in the EPx group (26.9% in the pre-COVID-19 period and 34.6% during the COVID-19 period), indicating greater strain diversity.

Assignment to a phylogenetic group and sequence type showed no statistical differences, as the majority of the ESBL-EC isolates from the pre-COVID-19 (n = 91; 76.5%) and COVID-19 (n = 59; 72.8%) periods were assigned to phylogenetic group B2. Although the percentage of isolates assigned to the ST131 sequence type group was slightly higher in the pre-COVID-19 period (n = 85; 71.4%) compared with the COVID-19 period (n = 55; 67.9%), the differences between the periods were not statistically significant (Table S3 in Supplementary Materials).

All isolates were tested for the presence of the 21 VAGs, with the only statistically significant difference detected for the genes *sat* (21.8% pre-COVID-19 versus 65.4% during COVID-19; *p* < 0.001) and *tsh* (0% pre-COVID-19 versus 4.9% during COVID-19; *p* = 0.014) (Table 4).

## 3. Discussion

The emergence and spread of ESBL-EC isolates pose a significant threat to public health. These bacteria exhibit resistance to a broad spectrum of antimicrobials, including β-lactams, SXT and fluoroquinolones, which are the cornerstone of therapy for numerous infections. Consequently, ESBL-EC infections can be difficult to treat, potentially leading to a high mortality rate [2].

Although AMR is an emerging problem, and the World Health Assembly adopted a global action plan on AMR in 2015, various antimicrobial agents, antivirals and anti-inflammatory drugs have been used for the effective treatment of COVID-19 patients [7,8,9,10]. In addition, during the COVID-19 pandemic, the use of antimicrobials, infection prevention measures through the use of biocides and changes in the healthcare system may have had an impact on the AMR burden [19,20,21].

When analyzing phenotypic resistance among ESBL-EC from the LRT, we observed a statistically significant decrease in FQ resistance in the COVID-19 period (from March 2020 to December 2022; n = 81) compared with the resistance data from the pre-COVID-19 period (from January 2017 to February 2020; n = 119) (Figure 1). Susceptibility to CIP and LVX increased by 11.4% (*p* = 0.019) and 10.6% (*p* = 0.033), respectively, during this period. Since ESBL-EC isolates from the LRT do not represent a specific pathotype but rather resemble other ExPEC isolates, we compared our data with data for other ExPEC strains, including those isolated from urinary tract infections and bloodstream infections. Our results for ESBL-EC from the LRT are in accordance with the percentage of fluoroquinolone-resistant invasive *E. coli* isolates retrieved from blood or cerebrospinal fluid samples, which decreased by 5.4% in 2022 in Slovenia compared with the European Accessibility Act (EU/EAA) range in 2022 [22]. These trends correspond with the declining consumption of quinolones both in the community (↓2.3%) and in the hospital sector (↓5.9%) between 2013 and 2022 in Slovenia [23] and are in line with the recommendations of the European Medicines Agency for the restricted use of FQ [24]. In addition, Abdelaziz Abdelmoneim et al. (2024) also detected a significant increase in *E. coli* susceptibility to quinolones in a comparative cross-sectional study in Egypt [25]. While the overall resistance rate to FQs between 2019 and 2022 in a study by Araújo et al. was similar to our results (i.e., 83.1% for Araújo et al. versus 81.5% in this study) in the same time period, they detected an increase in the resistance rate to FQs (ciprofloxacin and norfloxacin) between 2019 and 2022 among ESBL-EC strains isolated from outpatients with UTIs [26].

During the COVID-19 pandemic, we also detected a significantly lower resistance rate to non β-lactam antimicrobial SXT (Figure 1), a combination of trimethoprim and sulfamethoxazole commonly used for the treatment urinary tract, respiratory tract and gastrointestinal tract infections [12]. In our study, the resistance rate to SXT among ESBL-EC from the LRT in the pre-COVID-19 period was 76.5% (n = 91), and it decreased to 54.3% (n = 44) in the COVID-19 period. A similar proportion of SXT-resistant ESBL-EC (80%) isolated from UTIs between 2016 and 2019 (pre-COVID-19), was detected by Kettani Halabi et al. [27]. In contrast to our results, Abdelaziz Abdelmoneim et al. (2024) detected a significant increase in the resistance rate by 11.2% in *E. coli* between 2019 and 2022 in Egypt [25].

To gain insight into the population structure of *E. coli* between the two study periods, data for ARGs, ERIC profiles, phylogenetic groups, sequence type groups and VAGs were analyzed. Among the antimicrobial resistance genes, we confirmed significantly lower proportions of *bla*_TEM_, *aac(6′)-Ib-cr* and *qacEΔ1* in the COVID-19-period. In the COVID-19 period, CTX-M-2 group enzymes were detected for the first time among all ESBL-EC isolates from the LRT collected since 2002. The gene *bla*_CTX-M-2_ was detected in one isolate obtained from the LRT in 2021 and in one from 2022. In addition, a decrease in the most prevalent genes, *bla*_CTX-M-1_ (↓9.6%) and *bla*_OXA_ (↓9.9%), and an increase in *bla*_CTX-M-9_ (↑5.9%) were observed between the two study periods.

Comparing the ERIC-PCR profiles revealed a greater clonal diversity of ESBL-EC from the COVID-19 period. The most prevalent ERIC profile group, namely EP1, was statistically significantly associated with the pre-COVID-19 period (*p* = 0.001), while the EP4 group was related to the COVID-19 period (*p* = 0.012). Although we detected more clonally diverse isolates in the COVID-19 period, no statistical difference in phylogenetic and sequence type group assignments was detected. The majority of all ESBL-EC isolates (n = 200) were assigned to phylogenetic group B2 (n = 150; 75%) and sequence group ST131 (n = 140; 70%), which is a globally disseminated multidrug-resistant clone [28]. Therefore, the lower prevalence of ST131 in the COVID-19 period (↓3.5%), and the increased clonal diversity could be explained by the introduction of new bacterial strains, possibly due to a new sequence type (e.g., ST1193). According to a study by Pitout et al. (2022), ST1193 could account for up to 51% of FQ-resistant isolates and up to 40% of ESBL-producing *E. coli* isolates [29].

When comparing the prevalence of VAGs before and during the COVID-19 pandemic, only statistical increases in *sat* (*p* < 0.001) and *tsh* (*p* = 0.014) from the autotransporters group were detected. To compare our results for ARGs and VAGs with other studies, to the best of our knowledge, we could not find any similar reports. In addition, baseline patient data and sample types were compared between the two study periods, revealing a significantly higher proportion of bronchoalveolar lavage samples in the COVID-19 period (12.3%) compared with the pre-COVID-19 sample type (1.7%; *p* = 0.002). This observation of our study could be explained by the predominance of patients with severe COVID-19 in intensive care units requiring mechanical ventilation due to COVID-19-related acute respiratory distress syndrome [30].

## 4. Materials and Methods

### 4.1. Bacterial Strains and Patients

*E. coli* strains were isolated from LRT samples, including sputa, tracheal aspirates and bronchoalveolar lavages collected from patients hospitalized in various healthcare facilities in the Central Slovenia region. These facilities included a large university hospital, a national oncology center, a general hospital and several specialized outpatient and community healthcare centers. To compare ESBL-EC isolated in the pre-COVID-19 and COVID-19 periods, data obtained between 2017 and 2022 were analyzed. The dividing line between the two periods was March 2020 (isolates isolated in March 2020 were included in the COVID-19 period), as the Slovenian government declared an epidemic in Slovenia at that time [31,32] after the WHO declared a global pandemic on 11 March 2020 [33]. While data for ESBL-EC isolated in the pre-COVID period have been available [16], isolates from the COVID period (from January 2020 to December 2022) have been identified at the Institute of Microbiology and Immunology, Faculty of Medicine, University of Ljubljana (IMI) using matrix-assisted laser desorption/ionization time-of-flight mass spectrometry (MALDI TOF MS) with the MBT COMPASS 4.1 system (Microflex, Bruker Daltonics, Bremen, Germany) and analyzed as part of this and a biocide gene prevalence study [34].

### 4.2. Antimicrobial Susceptibility Testing

A disk diffusion assay was used to determine the phenotypic resistance for the following antimicrobial agents: ampicillin (AM; 10 µg), amoxicillin-clavulanic acid (AMC; 20–10 µg), piperacillin-tazobactam (TZP; 30–6 µg), cefuroxime (CXM; 30 µg), ceftazidime (CAZ; 10 µg), cefotaxime (CTX; 5 µg), ceftriaxone (CRO; 30 µg), cefepime (FEP; 30 µg), aztreonam (ATM; 30 µg), ertapenem (ETP; 10 µg), imipenem (IPM; 10 µg), meropenem (MEM; 10 µg), amikacin (AN; 30 µg), gentamicin (GM; 10 µg), trimethoprim-sulphametoxazole (SXT; 1.25–23.75 µg), ciprofloxacin (CIP; 5 µg) and levofloxacin (LVX; 5 µg). The results were interpreted according to European Committee on Antimicrobial Susceptibility Testing (EUCAST) guidelines [35]. Extended-spectrum β-lactamase production was tested according to the EUCAST’s recommendations [36]. A total of 86 consecutive, unduplicated, phenotypically positive ESBL-EC samples isolated from the COVID-19 period were subjected to further molecular analysis (Table S1 in Supplementary Materials). These isolates were classified as MDR based on the defined antimicrobial categories and antimicrobial agents for *Enterobacteriaceae*. MDR isolates were defined as non-susceptible to at least one agent in three or more antimicrobial categories. The following groups were considered individual antimicrobial categories based on the tested agents: penicillins (including AM), penicillins + β-lactamase inhibitors (including AMC), antipseudomonal penicillins + β-lactamase inhibitors (including TZP), non-extended spectrum cephalosporins (including CXM), extended-spectrum cephalosporins (including CAZ, CTX, CRO and FEP), monobactams (including ATM), carbapenems (including ETP, IPM and MEM), aminoglycosides (including AN and GM), folate pathway inhibitors (including SXT) and fluoroquinolones (including CIP and LVX) (Table S1 in Supplementary Materials) [37,38]. Isolates from the pre-COVID-19 period had been previously classified as MDR [16].

### 4.3. Molecular Characterization of ESBL-EC Isolates

Crude bacterial lysates were prepared using the boiling technique [39] and subsequently utilized for all PCR reactions. In brief, following DNA preparation, PCR reactions were conducted for antimicrobial resistance genes (*qnrA*, *qnrB*, *qnrS*, *qnrC*, *qnrD*, *qnrVC*, *aac(6′)-Ib-cr* and *qepA*) [40,41,42,43,44,45,46,47,48,49], ERIC profiles [50], phylogenetic group [51,52] and sequence type assignments [53], O25b typing [54] and virulence-associated genes (VAGs) ((adhesins: *afa/dra*, *fimH*, *iha*, *papC* and *papG*II; autotransporters: *fluA*, *sat*, *tsh* and *vat*; iron acquisition systems: *fyuA*, *iroN*, *irp2*, *iucD* and *iutA*; protectins: *iss*, *kpsMT*II, *ompT*_APEC_ and *traT*); and toxins (*hlyA*, *ehxA* and *usp*)) [16,55,56,57,58,59,60,61,62,63,64,65,66]. All PCR amplifications, except for phylogenetic group assignment according to the revised Clermont protocol [51] were performed in a total volume of 25 μL, containing 2 µL of the bacterial lysate, 12.5 μL of the PCR Master mix (Thermo Fisher Scientific, Waltham, MA, USA) and a 10 μM concentration of each primer. The primers and cycling conditions for these reactions are described in Table S2 in the Supplementary Materials. The selected antimicrobial genes were molecularly characterized in our previous study [34].

### 4.4. Statistical Analysis

Statistical analysis was conducted using IBM SPSS Statistics (version 25, IBM Analytics, NY, USA). Dichotomous variables were compared using Pearson’s Chi-squared test and described as frequencies and percentages. All tests were two-sided, with *p* values < 0.05 considered statistically significant and *p* values < 0.001 considered highly statistically significant. Pearson’s Chi-squared test was used to compare the results between the pre-COVID-19 (from January 2017 to February 2020; n = 119) and COVID-19 (from March 2020 to December 2022; n = 81) periods.

## 5. Conclusions

The available data on the impact of the COVID-19 pandemic on the epidemiology and antimicrobial resistance among the most frequent bacterial pathogens are often contradictory. While several studies have reported increased rates of multidrug resistant bacteria during the COVID-19 pandemic, other studies, including ours, have found increased susceptibility to certain groups of antimicrobials.

When analyzing the antimicrobial resistance patterns of ESBL-EC isolates from lower respiratory tract samples, we noticed a significantly decreased resistance rate to fluoroquinolones (FQs) and trimethoprim-sulfamethoxazole (SXT). A comparison of the ESBL-EC genotypes of strains from the COVID-19 period revealed a greater clonal diversity reflected in different percentages of resistance and virulence-associated genes. It should be considered that changes in healthcare practices and patient demographics during the COVID-19 pandemic could have distorted the observed changes in resistance patterns. Consequently, the changed population structure of ESBL-EC could be related to the emergence and survival of novel patient-associated bacterial strains with distinct genotypes from the community together with more stringent measures in hospital settings, thus preventing the spread of (competing) hospital-associated strains. Further genomic studies encompassing a broader range of strains and locations could shed light on the underlying drivers and mechanisms behind the restructuring of these bacterial populations.

## Figures and Tables

**Table 1:** Demographic and clinical sample data for ESBL-EC isolated before and during the outbreak of the COVID-19 pandemic.

	Pre-COVID-19 N = 119 (100%) n (%)	COVID-19 N = 81 (100%) n (%)	Pearson’s Chi-Squared Value (df 1)	*p* Value ^1^
Average age	67.3	70.8	/	/
0–10 years	11 (9.2%)	2 (2.5%)	3.639	0.056
11–20 years	1 (0.8%)	0 (0%)	0.684	0.408
21–30 years	1 (0.8%)	1 (1.2%)	0.076	0.783
31–40 years	0 (0%)	1 (1.2%)	1.477	0.224
41–50 years	7 (5.9%)	5 (6.2%)	0.007	0.932
51–60 years	7 (5.9%)	5 (6.2%)	0.007	0.932
61–70 years	20 (16.8%)	18 (22.2%)	0.918	0.338
71–80 years	28 (23.5%)	20 (24.7%)	0.036	0.85
81–90 years	36 (30.3%)	27 (33.3%)	0.212	0.645
91+ years	8 (6.7%)	2 (2.5%)	1.836	0.175
Male	70 (58.8%)	57 (70.4%)	2.772	0.096
Female	49 (41.2%)	24 (29.6%)	2.772	0.096
Tracheal aspirate	87 (73.1%)	50 (61.7%)	2.893	0.089
Sputum	30 (25.2%)	21 (25.9%)	0.013	0.909
Bronchoalveolar lavage	2 (1.7%)	10 (12.3%)	9.72	0.002

^1^ The *p* values (pre-COVID-19 vs. COVID-19) calculated by the Chi-squared test are shown, where *p* values < 0.05 were considered statistically significant and *p* values < 0.001 were considered highly statistically significant.

**Table 2:** Prevalence of antimicrobial and integron-associated genes of ESBL-EC before and during the outbreak of the COVID-19 pandemic.

	Pre-COVID-19 N = 119 (100%) n (%)	COVID-19 N = 81 (100%) n (%)	Pearson’s Chi-Squared Value (df 1)	*p* Value ^1^
Plasmid-mediated quinolone resistance genes				
*qnrA*	0 (0%)	0 (0%)	/	/
*qnrB*	0 (0%)	0 (0%)	/	/
*qnrS*	3 (2.5%)	4 (4.9%)	0.834	0.361
*qnrC*	0 (0%)	0 (0%)	/	/
*qnrD*	0 (0%)	0 (0%)	/	/
*qnrVC*	0 (0%)	0 (0%)	/	/
*qepA*	0 (0%)	0 (0%)	/	/
*aac(6′)-I-cr*	46 (38.7%)	20 (24.7%)	4.25	0.039
β-lactamase group genes				
*bla*_CTX-M-1_	101 (84.9%)	61 (75.3%)	2.865	0.091
*bla*_CTX-M-2_	0 (0%)	2 (2.5%)	2.968	0.085
*bla*_CTX-M-9_	15 (12.6%)	15 (18.5%)	1.322	0.25
*bla*_CTX-M-8_	0 (0%)	0 (0%)	/	/
*bla*_CTX-M-25_	0 (0%)	0 (0%)	/	/
*bla*_TEM_	63 (52.9%)	26 (32.1%)	8.477	0.004
*bla*_SHV_	1 (0.8%)	0 (0%)	0.684	0.408
*bla*_OXA_	47 (39.5%)	24 (29.6%)	2.049	0.152
Biocide resistance genes encoded on mobile genetic elements				
*qacEΔ1*	62 (52.1%)	24 (29.6%)	9.929	0.002
*qacE*	0 (0%)	0 (0%)	/	/
*qacF/H/I*	0 (0%)	1 (1.2%)	1.477	0.224
*qacG*	0 (0%)	0 (0%)	/	/
*sugE* (p)	0 (0%)	0 (0%)	/	/
Chromosome-encoded biocide resistance genes				
*emrE*	92 (77.3%)	65 (80.2%)	0.246	0.62
*mdfA*	119 (100%)	81 (100%)	/	/
*sugE* (c)	119 (100%)	81 (100%)	/	/
*ydgE*	119 (100%)	81 (100%)	/	/
*ydgF*	119 (100%)	81 (100%)	/	/
Integrons				
*int1*	33 (27.7%)	37 (45.7%)	6.824	0.009
*int2*	0 (0%)	1 (1.2%)	1.477	0.224
*int3*	0 (0%)	0 (0%)	/	/

^1^ The *p* values (pre-COVID-19 vs. COVID-19) calculated by a Chi-squared test are shown, where *p* values < 0.05 were considered statistically significant and *p* values < 0.001 were considered highly statistically significant.

**Table 3:** Clonal diversity of ESBL-EC isolated before and during the COVID-19 pandemic according to ERIC-PCR profile groups.

	Pre-COVID-19 N = 119 (100%) n (%)	COVID-19 N = 81 (100%) n (%)	Pearson’s Chi-Squared Value (df 1)	*p* Value ^1^
EP1	54 (45.4%)	18 (22.2%)	11.216	0.001
EP2	8 (6.7%)	0 (0%)	5.672	0.017
EP3	21 (17.6%)	23 (28.4%)	3.244	0.072
EP4	3 (2.5%)	9 (11.1%)	6.306	0.012
EP5	1 (0.8%)	1 (1.2%)	0.076	0.783
EP6	0 (0%)	2 (2.5%)	2.968	0.085
EPx	32 (26.9%)	28 (34.6%)	1.353	0.245

^1^ The *p* values (pre-COVID-19 vs. COVID-19) calculated by a Chi-squared test are shown, where *p* values < 0.05 were considered statistically significant and *p* values < 0.001 were considered highly statistically significant.

**Table 4:** Prevalence of virulence-associated genes of ESBL-EC isolated in the periods before and during the outbreak of the COVID-19 pandemic.

	Pre-COVID-19 N = 119 (100%) n (%)	COVID-19 N = 81 (100%) n (%)	Pearson’s Chi-Squared Value (df 1)	*p* Value ^1^
Adhesins				
*afa/dra*	31 (26.1%)	13 (16%)	2.809	0.094
*fimH*	115 (96.6%)	77 (95.1%)	0.312	0.576
*iha*	86 (72.3%)	50 (61.7%)	2.461	0.117
*papC*	15 (12.6%)	14 (17.3%)	0.851	0.356
*papG*II	12 (10.1%)	11 (13.6%)	0.579	0.447
Autotransporters				
*fluA*	88 (73.9%)	64 (79%)	0.677	0.411
*sat*	26 (21.8%)	53 (65.4%)	38.308	<0.001
*tsh*	0 (0%)	4 (4.9%)	5.996	0.014
*vat*	4 (3.4%)	5 (6.2%)	0.886	0.346
Protectins				
*iss*	12 (10.1%)	12 (14.8%)	1.021	0.312
*kpsMT*II	81 (68.1%)	55 (67.9%)	0.001	0.98
*ompT*_APEC_	14 (11.8%)	13 (16%)	0.758	0.384
*traT*	98 (82.4%)	69 (85.2%)	0.281	0.596
Iron acquisition systems				
*fyuA*	105 (88.2%)	68 (84%)	0.758	0.384
*iroN*	15 (12.6%)	13 (16%)	0.475	0.491
*irp2*	107 (89.9%)	69 (85.2%)	1.021	0.312
*iucD*	97 (81.5%)	70 (86.4%)	0.842	0.359
*iutA*	98 (82.4%)	70 (86.4%)	0.593	0.441
Toxins				
*ehxA*	0 (0%)	0 (0%)	/	/
*hlyA*	14 (11.8%)	9 (11.1%)	0.02	0.887

^1^ The *p* values (pre-COVID-19 vs. COVID-19) calculated by a Chi-squared test are shown, where *p* values < 0.05 were considered statistically significant and *p* values < 0.001 were considered highly statistically significant.

## Data Availability

The data supporting the results of this study are available in the Supplementary Materials or upon reasonable request from the corresponding author (J.A.A.).

## Supplementary Materials

The following supporting information can be downloaded at https://www.mdpi.com/article/10.3390/antibiotics13090797/s1. Table S1: Research raw data; Table S2: Primers and conditions for PCR amplification; Table S3: Comparison of phylogenetic group and sequence type assignment of the ESBL-EC isolates isolated in the periods before and during the outbreak of the COVID-19 pandemic; Table S4: Prevalence of antimicrobial resistance among ESBL-EC isolates from LRT; Table S5: Resistance profiles of 200 ESBL-EC isolates.

## Abbreviations

AM = ampicillin; AMC = amoxicillin-clavulanic acid; AMR = antimicrobial resistance; AN = amikacin; ARGs = antimicrobial resistance genes; ATM = aztreonam; CAZ = ceftazidime; CIP = ciprofloxacin; CLSI = Clinical and Laboratory Standards Institute; COVID-19 = during COVID-19 pandemic period; CRO = ceftriaxone; CTX = cefotaxime; CTX-M = cefotaxime-hydrolyzing β-lactamase–Munich; CXM = cefuroxime; ERIC-PCR = enterobacterial repetitive intergenic consensus polymerase chain reaction; ESBL = extended-spectrum β-lactamase; ESBL-EC = extended-spectrum β-lactamase-producing *E. coli*; ETP = ertapenem; EUCAST = European Committee on Antimicrobial Susceptibility Testing; EU/EAA = European Accessibility Act; ExPEC = extraintestinal pathogenic *E. coli*; FEP = cefepime; FQs = fluoroquinolones; GM = gentamicin; IMI = Institute of Microbiology and Immunology, Faculty of Medicine, University of Ljubljana; IPM = imipenem; LVX = levofloxacin; LRT = lower respiratory tract; LRTI = lower respiratory tract infection; MALDI TOF MS = matrix-assisted laser desorption/ionization time-of-flight mass spectrometry; MDR = multidrug-resistant; MEM = meropenem; OXA = oxacillinases; pre-COVID-19 = before COVID-19 pandemic period; SHV = sulfhydryl variant of the TEM enzyme; ST1193 = sequence type 1193; ST131 = sequence type 131; SXT = trimethoprim-sulphametoxazole; TEM = Temoneira class A extended-spectrum β-lactamase; TZP = piperacillin-tazobactam; UTIs = urinary tract infections; VAGs = virulence-associated genes; WHO = World Health Organization.
